# Autonomous patient consent for anaesthesia without preoperative consultation: a qualitative feasibility study including low-risk procedures

**DOI:** 10.1016/j.bjao.2022.100022

**Published:** 2022-07-13

**Authors:** Marije Marsman, Wisse M.F. van den Beuken, Wilton A. van Klei, Teus H. Kappen

**Affiliations:** 1 Department of Anaesthesiology, University Medical Center Utrecht, Utrecht, the Netherlands; 2 Department of Anesthesia and Pain Management, Toronto General Hospital, University Health Network Toronto, Toronto, ON, Canada; 3 Department of Anesthesiology and Pain Medicine, Temerty Faculty of Medicine, University of Toronto, Toronto, ON, Canada; 4 Toronto General Hospital Research Institute, Toronto, ON, Canada; 5 Department of Information Technology, University Medical Center Utrecht, Utrecht, the Netherlands

**Keywords:** application, autonomy, digitisation, e-health, empowerment, information provisioning, informed consent

## Abstract

**Background:**

Informed consent for anaesthesia is mandatory and requires provision of information and subsequent consent during consultation between anaesthesiologist and patient. Although information can be provided in an electronic format, it is unknown whether this a valid substitute for a consultation. We explored whether provision of digital information is equivalent to oral consultation and whether it enables patients to give electronic informed consent (e-consent) for anaesthesia.

**Methods:**

Qualitative feasibility study using semi-structured interviews in 20 low-risk adults scheduled for minor surgery under general anaesthesia or procedural sedation at a university hospital. Data were analysed using a thematic content analysis approach. During the interviews, patients followed an application that provides information and subsequent e-consenting.

**Results:**

The mean age was 50 yr and patients had good digital skills. Fifteen patients (75%) had previous experience of anaesthesia. The digital application provided enough information for all patients, but eight (40%) preferred consultation with an anaesthesiologist, mainly for personal contact. Patients had different information needs, with previous experiences leading to lower information needs. Nineteen patients had sufficient information to consent autonomously. Most patients considered separate anaesthesia consent superfluous to the surgical consent.

**Conclusion:**

The digital application provided sufficient information and patients valued the information offered and the advantage of processing information at their own pace. This information made patients feel empowered to autonomously consent to anaesthesia without consultation. Remarkably, consent for anaesthesia was considered unimportant, because patients felt they had ‘no choice’ if they wanted to undergo surgery.

According to current guidelines, patients must give informed consent for anaesthesia, in addition to their consent for surgery.^1^, ^2^, ^3^, ^4^, ^5^ From a medical perspective this separation of consents makes sense: anaesthesia is a procedure distinct from the surgical procedure, with its own risks and complications. Many countries require anaesthesiologists to provide information about anaesthesia, to guide patients in their deliberation about risks and benefits of anaesthesia and to obtain consent for anaesthesia during a consultation.^1^^,^^2^^,^^5^ For example in The Netherlands, verbal consent needs to be given to the anaesthesiologist and this should be noted in the medical record. However, consent for anaesthesia is not as straightforward as it seems.

In general, consenting requires patients to weigh risks and benefits of the proposed intervention. As anaesthesia is not an isolated intervention, but an inevitable component of the main treatment, that is the surgery, patients have to weigh the risks of anaesthesia against the benefits of the surgical procedure. However, most patients will have weighed surgical risks and consented to the planned surgical procedure before they visit the anaesthesiologist. As anaesthesiologists may not be fully aware of the risks and benefits of the surgical procedure, this can make guidance of anaesthesia risks related to surgical risks and benefits suboptimal.

The value of anaesthesiologists as providers of information also remains uncertain, even though many authors consider it to be important.^2^^,^^6^, ^7^, ^8^, ^9^ Patients require information that is tailored to their individual needs, which they can understand and remember, but research has shown that information offered by physicians is often too complex or biased.^9^, ^10^, ^11^, ^12^, ^13^ Therefore, instead of relying on a verbal consultation with a physician, other ways may be more effective in informing patients, for example using multimedia. Studies have shown that provision of multimedia-based information about a procedure is equal – or even superior – to provision of information by a physician in terms of reducing anxiety and knowledge retention.^14^, ^15^, ^16^, ^17^, ^18^, ^19^, ^20^ Especially when decision-support applications guide patients in their informational needs before the informed consent consultation with physicians, patients seem to be better informed.^12^^,^^16^^,^^21^

If anaesthesiologists cannot fully guide the informed consent process, then provision of adequate information could be sufficient for patients to provide informed consent for anaesthesia. Moreover, if information offered digitally is equivalent to information provided by an anaesthesiologist, then patients may not need a consultation. We therefore hypothesised that an interactive web-based digital application could offer sufficient information about anaesthesia to make patients feel well informed. We additionally hypothesised that this information could empower them to give their consent digitally (e-consent) without having consulted an anaesthesiologist.

## Methods

### Study design and setting

This study was conducted at the pre-anaesthesia assessment clinic at the University Medical Center Utrecht, a tertiary referral hospital in The Netherlands. We used semi-structured interviews to answer four research questions:

1. Are patients able to independently select and process information about anaesthesia without consulting an anaesthesiologist (autonomous information provision)?
2. Can a digital application provide sufficient information for informed consent equivalent to verbal consultation?
3. Do patients feel empowered to give autonomous consent, that is consent without consulting an anaesthesiologist, after having received that information?
4. Under what conditions could provision of only digital information, e-consenting, or both be feasible?

After constructing our hypothesis based on the literature and expert opinion, one author (MM) interviewed seven adults, not scheduled for anaesthesia, in open, exploratory interviews. The goal was to obtain information about the patient perspective regarding content of the desired application to be developed, to determine the domain in which our research questions could be explored, and to test interview questions. We concluded that provision of digital information and electronic informed consent (e-consent) could be explored in relatively healthy patients (ASA 1 and 2) scheduled for minor procedures, with a low risk for complications. A framework was constructed for the web-based digital application and for a semi-structured interview guide (Appendix A). The interview guide was subsequently pilot-tested twice in healthy adults who were not scheduled for surgery, and questions were refined.

### Digital application with e-consent

The digital e-consent application is a prototype of an interactive web-based programme. Every web page provides basic information around a theme, such as ‘what is procedural sedation’, using both text and video (Figure 1 online video and Appendix A). Patients can receive additional information by selecting a subtitle on a page. Subsequently, patients can mark the checkbox if they have understood the information. The application ends with marking the informed consent checkbox. If patients feel they cannot consent or if they have questions, they mark the ‘no’ or ‘doubt’ checkbox, which results in an appointment for a consultation.

Supplementary video related to this article can be found at https://doi.org/10.1016/j.bjao.2022.100022

The following is the supplementary data related to this article:

Video 1 2 Video 1

### Patients

Eligible patients were low surgical-risk adults (ASA 1 and 2) scheduled for elective minor procedures under either general anaesthesia or procedural sedation and able to speak and read the Dutch language. Patients scheduled for a pre-anaesthesia consultation at the outpatient clinic were screened for eligibility based on our inclusion criteria. After the interview, patients had their regular pre-anaesthesia consultation. Patients were interviewed between March and June 2020. After interviewing the 15th patient, five patients were included because they were younger or had no experience of anaesthesia, after which no new insights were obtained regarding provision of information and consent (data saturation was achieved).

### Interview

One author (WB) conducted all interviews, which were recorded by video. Baseline data were obtained using a short questionnaire (Appendix B). No data were collected from the medical record. Patients followed the digital application, while the interviewer asked questions according to the interview guide (Appendix A) and observed reading behaviour. Between interviews, questions were adjusted if necessary, based on topics mentioned by previous patients and analysis of previous interviews.

### Ethics

The Medical Research Ethics Committee of the UMC Utrecht waived the need for informed consent (20–117/C), but all patients consented to the use of personal data for the purpose of the study under the General Data Protection Regulation (GDPR).

### Data analysis

The recorded interviews were transcribed *verbatim* and anonymised. We used a thematic content analysis strategy, in which data analysis is a circular process and interlinks with data collection. An initial coding framework was created, consisting of deductive and inductive codes. WB coded transcripts 1 to 10 and MM 11 to 20. The coding framework was continuously adjusted with analysis of every subsequent interview by rereading, discussing, and recoding previous interviews (Appendix C). MM and WB cross-checked several transcripts for the created codes, and if there were discrepancies a discussion followed until agreement was reached. Next, codes were compared, categorised, and combined with patient characteristics leading to development of concepts around the research questions and identification of overlapping themes. Baseline data from the short questionnaire are presented as frequencies with percentages. In addition, some (open) questions from the interview guide resulted in answers that were quantifiable, because interviewees gave comparable answers.

Nvivo 12 pro was used for coding.

## Results

Twenty patients were interviewed (interviewees), of whom 18 were interviewed before their pre-anaesthesia consultation and two immediately after, owing to logistics. The mean age was 50 (range, 20–75) yr and 12 were female (Table 1). All interviewees had good digital skills and 15 (75%) accessed their online electronic health records regularly. Fifteen interviewees (75%) had already undergone their planned type of anaesthesia during a previous procedure (experienced interviewees) (Table 1).

**Table 1:** Baseline characteristics. Data shown are numbers (%) of patients, unless stated otherwise. ∗Digital skills included use of computer for leisure, social media, buying goods, work, e-mail, taxes, insurance, private banking, seeking information about own health, online access to personal hospital heath record. Use of a computer for <4 activities was regarded as low digital skills, 4–8 as intermediate, and >8 as good. ^†^Various surgeries were performed by the different specialties. For example, endoscopy: oesophageal dilation; cardiology: ablation for arrhythmia; general/orthopaedics: knee arthroscopy and removal of plates; gynaecology: laparoscopic procedures; ENT/plastic: changing breast prosthesis, nose surgery; ophthalmology: strabismus surgery, eyelid corrections, vitrectomy.

Baseline		Number of patients (%)
Female sex (%)		12 (60)
Age categories (yr)		
	20–39	4 (20)
	40–59	11 (55)
	60–79	5 (25)
Highest education	High school	3 (15)
	Community college	8 (40)
	University	9 (45)
Digital skills∗	<4 (low)	0
	4–8 (intermediate)	5 (25)
	>8 (good)	15 (75)
Did patient seek information before anaesthesia consultation?	Yes	5 (25)
	No	15 (75)
How much did the patient want to know about anaesthesia?	Nothing	1 (5)
	As less as possible	0
	Enough to make a decision	16 (80)
	As much as possible	3 (15)
Previous experience with anaesthesia	Yes, conscious sedation	1 (5)
	Yes, general anaesthesia	10 (50)
	Yes, neuraxial anaesthesia	0
	Yes, peripheral nerve block	1 (5)
	Yes, ≥ 2, of above	5 (25)
	No	3 (15)
Scheduled anaesthesia technique	General anaesthesia	18 (90)
	Procedural sedation	2 (10)
Surgical speciality^†^	Endoscopy	1 (5)
	Cardiology	1 (5)
	General/orthopaedics	3 (15)
	Gynaecology	5 (25)
	ENT/plastic	3 (15)
	Ophthalmology	7 (35)

**Table 2:** Quotes from the interviews.

Quote no.	Patient	Topic	Quote
1	5	Need for information – experience	Last time I had no pain or sore throat … No I don't have this.
2	5	Need for information – trust in health care	I'm in good hands, you will do your best. I'll get anaesthesia and afterwards I wake up.
3	2	Information provisioning – severe complications	It's the same as reading a package leaflet, you already get sick just by reading it. So I just don't do it.
4	15	Information provisioning – severe complications	I have seen this [awareness] in a movie once, so you don't want this to happen to you.
5	2	Need for information – unexperienced	I have heard this 10 times before. The first time I read everything and asked questions.
6	16	Information provisioning – severe complications	My brother-in-law had complications, so I want to know what can go wrong even if it [the risk] is 1:100.
7	6	Information provisioning – severe complications – weighing information	These are just complications that can happen, but … crossing the street, there is also a change you get hit by a bus …
8	19	Informed consent – no choice	When I want to undergo that surgical procedure, I need to have the anaesthesia. So do I have a choice? Not really.
9	7	Informed consent – influence of severe complication	I just want that surgery, so I just have to accept the consequences.
10	8	Informed consent – consent surgery includes consent anaesthesia	To my opinion, it actually belongs together
11	18	Informed consent – unimportance anaesthesia	Consultation with the surgeon: yes. [Consultation with an] anaesthesiologist? I would fill it in [online] right away. No need to come to the hospital.
12	7	Informed consent – no need because of good health	No, for me there is no special indication to speak an anaesthesiologist. Everything is normal.
13	8	e-Consent *vs* anaesthesiologist – ‘undefined feeling’	It's about your health and you get general anaesthesia, I must have the feeling I'm safe.
14	4	e-Consent *vs* anaesthesiologist – personal contact	You can look into someone's eyes.
15	4	e-Consent *vs* anaesthesiologist – personal contact	It's about your life and that's important enough for me to have a personal consultation.
16	13	e-Consent *vs* anaesthesiologist – anxiety and asking questions	Personal contact …, perhaps relieves anxiety, you can ask extra questions. Perhaps you can be comforted.
17	3	e-Consent *vs* anaesthesiologist – medical safety	It would be strange to give informed consent … without physical examination.
18	20	e-Consent *vs* anaesthesiologist – high-risk surgery	I think that with heart surgery [the risk] is higher, so it would be great to speak to a physician to obtain more insight about [the risk] really being okay.
19	5	e-Consent *vs* anaesthesiologist – time saving	You are at home and can take your time reading. With extra [mouse] clicks you get extra information before you give an answer.
20	15	e-Consent *vs* anaesthesiologist – information provisioning	You have the choice: do I want more information or not.
21	13	e-Consent *vs* anaesthesiologist – information provisioning	[People] who choose digital are like: ‘I'll just see what'll happen’ or just don't want to give it to much attention.

### Research questions 1 and 2: receiving adequate digital information

After finishing the application, all interviewees felt they had received sufficient information. One interviewee (no previous anaesthesia) had a remaining question for an anaesthesiologist. The interviewees reported being capable of selecting and processing the information they wanted to receive.

We observed that interviewees differed in how they processed the information offered. Most interviewees did not read all the text. A frequently reported reason for not reading information was familiarity with the topic because of previous experience with anaesthesia (Table 2, quote 1). Some did not read additional information when they considered the subject irrelevant to their situation. Others preferred not to know too many details and reported to rely on the healthcare professionals (Table 2, quote 2), especially for severe complications (Table 2, quote 3).

Contrastingly, several reasons for reading information were reported. Some interviewees indicated that knowing what might happen to them was important, even concerning rare, yet severe, complications (Table 2, quote 4). Interviewees with no previous experience of anaesthesia read more, which was acknowledged by some interviewees with previous experience of anaesthesia who said they had required more information before their first experience of anaesthesia (Table 2, quote 5). We observed that patients who read additional information often reported previous negative experiences, such as side effects or complications on that particular topic. These experiences could be their own, but also those of others, such as family members (Table 2, quotes 4 and 6). After having read the risks, most patients considered the benefits of the planned procedure to outweigh the risks (Table 2, quote 7).

### Research question 3: empowered autonomous consent

We asked interviewees if they felt sufficiently informed to provide e-consent without consulting an anaesthesiologist. All interviewees felt empowered to give e-consent.

In addition, most interviewees reported that consent for anaesthesia itself was superfluous, as they felt they did not have a choice: their planned procedure would not be possible without anaesthesia (Table 2, quote 8). Several observations supported that patients had no need for a formal anaesthesia consent. Reading about side effects or rare severe complications did not make interviewees reassess their choice for anaesthesia (Table 2, quote 9). Also, we asked interviewees at different points within the application whether they could give informed consent at that point. Sixteen (80%) said they would already give consent at the first page with general information about anaesthesia, because of their previous experiences. We also observed that more than half of the interviewees thought that informed consent for surgery also included consent for anaesthesia (Table 2, quote 10). Consent for anaesthesia or consultation with an anaesthesiologist was not seen as contributory, because some interviewees regarded anaesthesia as less important than surgery (six interviewees, 30%, Table 2, quote 11). Some interviewees also reported (perceived) that the safety of anaesthesia was a reason for not needing to consent, because of their good health or low-risk procedure (Table 2, quote 12).

### Research question 4: feasibility of a digital application

Although all interviewees said they were well informed by the application, when offered the choice, eight interviewees (40%) preferred a consultation with an anaesthesiologist. The main reported reasons were need for personal contact (Table 2, quotes 13–16), the possibility to ask additional questions (Table 2, quote 16), and the perceived feeling that physical examination was necessary for a preoperative assessment (Table 2, quote 17). Some interviewees could not explain their preference for a consultation and reported it was more about ‘feeling safe’ (Table 2, quotes 13, 14). However, if the hospital were to ask them to only use the application, seven of the eight interviewees who preferred a consultation would accept a digital solution without a consultation, because they recognised that this could make healthcare more efficient. Only one interviewee would always choose a consultation, because of personal contact (Table 2, quotes 14 and 15).

### Information provisioning by consultation vs application: overall and patient-specific indications and benefits

The social aspect of a consultation was suggested as an important benefit and was reported by 14 interviewees (70%) for reasons of personal contact, comfort, and relieving anxiety (Table 2, quote 16). The option to ask additional questions and to obtain information tailored to specific individual needs was deemed important (reported by eight interviewees [40%]). Therefore, a consultation was suggested as appropriate for anxious or patients with no previous experience of anaesthesia, for patients with extra questions, low digital skills, or illiteracy, and for patients with comorbidities or major surgery to discuss patient- or procedure-specific risks (Table 2, quote 18) (Table 3).

Time was the most frequently reported benefit of using the digital application (Table 3). It saves traveling and waiting time (reported by 14 interviewees, 70%), and it allows patients to process the information at their own pace (11 interviewees, 55%, Table 2, quote 19). Seven interviewees considered an application advantageous, because provision of information can be tailored to their personal needs (Table 2, quotes 19 and 20). Interviewees reported that simple language and the combination of text and video made the information comprehensible. Interviewees thought electronic provision of information and e-consenting was suitable for patients with adequate digital skills, for patients in good health or undergoing minor procedures, for patients with previous experience of anaesthesia, and also for patients who did not want to receive much information (Table 2, quote 21) (Table 3).

**Table 3:** Comparison of application with consultation with anaesthesiologist.

Application with e-consent		Consultation with anaesthesiologist	
Benefit	Indication	Benefit	Indication
Time	Social	Social	Social
*Patient perspective*	Experienced	Personal contact	Unexperienced
Traveling time	Uninterested	Comforting	Anxious patients
Waiting time	No questions	Reducing anxiety	Need for personal contact
Following application any time of day	‘Not wanting to know’		
Own pace			
	**Medical**	**Medical**	**Medical**
*Anaesthesiologist perspective*	Good health	Physical examination	High-risk procedure
More time for other duties	Low-risk procedure	Anamnesis	Comorbidities
	**Communication**	**Safety**	**Communication**
	Digitally skilled	No data leak	Low digital skills
	Young patients		Older patients
			Other language
**Information provisioning**		**Information provisioning**	
Video		Answering extra questions by anaesthesiologist or patient	
Simple language			
Consistency in information ordered			
Autonomy what information to read			

Age was reported by the interviewees as a potentially complicating factor. Generally, younger patients were seen as digitally skilled compared with older patients, and applications were therefore regarded as suitable for younger patients and consultation for older patients. However, after asking for an age limit, interviewees reconsidered and indicated it would depend on individual digital skills.

## Discussion

In this qualitative study, we investigated whether a digital application provided sufficient information to healthy adults scheduled for minor procedures under general anaesthesia or procedural sedation compared with a consultation and if so, if patients would be able to autonomously consent to anaesthesia. Patients felt well informed, valued the information offered, and appreciated the autonomy provided by the application. They felt empowered to consent without consulting an anaesthesiologist. Most interestingly, consenting for anaesthesia was considered unimportant, because patients felt they had ‘no choice’ if they wanted to undergo surgery.

In this study the patients reported that they would give an e-informed consent. Interestingly, most patients felt no need to consent for anaesthesia at all, because they already had consented to surgery and considered this inseparable from anaesthesia. The complex relation between both consents has been discussed before.^8^, ^9^, ^10^^,^^13^^,^^22^ Historically, consents were separated for medical and legal reasons, as there was need for provision of information and risk assessment related to anaesthesia by anaesthesia experts.^6^ Despite these reasons for a separate consent, this study shows this separation is unhelpful for patients. As patients have to consider risks for the whole ‘perioperative package’, separating anaesthesia and surgery seems artificial. From a patient perspective, it seems more logical to combine surgical and anaesthesia education and risk assessment, and to give a single ‘perioperative consent’. For low-risk procedures and patients, a digital application about anaesthesia could be an option for timely provision of information about the anaesthetic part of this consent.

Patients valued provision of information as very important in the consent process. This has been acknowledged in a study by Fung and Cohen.^6^ We observed major differences in the quantity of information patients read, but all patients were satisfied with the amount of information. In our application, patients had the autonomy to choose how much information to read, how to receive this information (text, video, or both), and when to read it, which was regarded as superior to verbal information from a physician. A study by van den Berg and colleagues^12^ on shared decision-making for postoperative analgesia also showed that anaesthesiologists did not tailor information to the needs of the patient and that a decision support tool would provide added knowledge. Current reviews and editorials about informed consent emphasise the importance of provision of patient tailored information, but acknowledge that this is very difficult for physicians.^8^^,^^9^^,^^13^^,^^22^ If information meets the patient's needs this will be more likely to result in a satisfied patient.^8^^,^^9^^,^^13^^,^^22^ A digital application may be more likely to fulfil the informational needs of individual patients.

In this study, 15 patients (75%) had previous experience of their planned anaesthesia technique. This previous experience lowered their informational need, because patients trusted their previous experiences to be also applicable to their upcoming anaesthesia.^18^^,^^23^ Patients without previous experience indeed read more information. This observation adds to the conflicting literature regarding the influence of previous experience on information needs.^14^^,^^24^

This study has several strengths. The interviews offered a broad insight into patient's perceptions about provision of information and consent. Thanks to the cyclic process of data gathering and analysis, newly evolved themes during interviews could be explored in subsequent interviews. The application gave us real-time insight into the patients' collection and processing of information, which reduced the chances of recollection bias.

Our study also has some limitations. First, qualitative research can be prone to interpretation bias, during coding and analysis. We reduced this by cross-checking codes and discussing if in doubt. Also, the results from the analysis were discussed by three researchers (MM, WB, TK). Second, 20 patients were interviewed but this number is in line with current qualitative research studies in which thoughts and perceptions from patients about a specific topic are explored. Based on our exploratory interviews, we chose a restricted domain to explore our hypotheses: low-risk patients undergoing minor procedures, thus limiting the generalisability of our study.

### Implications for current practice and future studies

Digital applications have the potential to replace in-person provision of information by an anaesthesiologist for low-risk patients scheduled for minor procedures under procedural sedation or general anaesthesia. In The Netherlands, all patients have a pre-anaesthesia consultation at the outpatient clinic days to weeks before their procedure, making the use of digital applications as a replacement for consultations potentially cost-effective. For healthcare systems with a pre-anaesthesia consultation at the day of the procedure, digital applications could be offered in advance, making patients better prepared for their procedures. This could replace the consultation on the day of the procedure or reduce the consultation time, making healthcare more cost-effective. It is likely that for other, more complex patients, use of an application could add to a patient's knowledge ahead of a consultation and result in a more in-depth discussion during the consultation.^12^^,^^16^^,^^17^ Digital applications offer patients autonomy in time management and acquisition of information, which will probably result in higher patient satisfaction compared with traditional consultations. Upcoming research should explore how information should be offered to patients, adjusted to individual needs.^14^^,^^16^^,^^17^^,^^19^ The patient's perceptions about provision of information in other domains should also be investigated, such as in those planned for major surgery or when patients have to choose between anaesthetic procedures. Lastly, a focus should be on appropriate selection of patients capable of using digital applications as our study shows that some patients need a consultation.

We also studied the concept of autonomous informed consent. Consent is an important right of patients, and current laws require anaesthesiologists to be ‘physically’ involved in this process. However, this study has shown that some patients feel empowered to consent autonomously, provided that they received adequate information. As digitisation and autonomy are rapidly expanding in society and medicine, it is important to test if such an autonomous informed consent could be possible under certain conditions. Our ‘proof-of-concept’ could serve as a framework for further research on informed consent and stimulate the adaptation of guidelines and laws to societal changes.

It may also be worth exploring the possibility of a ‘common informed consent’ in which anaesthesiologists and surgeons work together in provision of (digital) information and subsequently obtaining a single consent for the whole perioperative process.

## Conclusion

A digital application provided sufficient information to healthy adults scheduled for minor procedures under general anaesthesia or procedural sedation and offered patients the advantage of processing information at their own pace and well ahead of the planned procedure. This information empowered patients to autonomously consent to anaesthesia without consulting an anaesthesiologist. From the patient's perspective, consenting for anaesthesia separately from surgery was considered complex and unnecessary, but receiving sufficient information about anaesthesia to get confidence in a good outcome was considered important.

## Authors' contributions

Substantial contribution to conception and design, acquisition of data, or analysis and interpretation of data: MM, WB, WK, TK.

Drafting the article or revising it critically for important intellectual content: MM, WB, WK, TK.

Final approval of the version to be published: MM, WB, WK, TK.

Agreement to be accountable for all aspects of the work thereby ensuring that questions related to the accuracy or integrity of any part of the work are appropriately investigated and resolved: MM, WB, WK, TK.

## Declarations of interest

The authors declare that they have no conflicts of interest.

## Funding

Department of Anaesthesiology, University Medical Center Utrecht, Utrecht, The Netherlands.

## References

[1] American Association of Nurse Anesthetists (2016). https://www.aana.com.

[2] De Hert S., Staender S., Fritsch G. (2018). Pre-operative evaluation of adults undergoing elective noncardiac surgery: updated guideline from the European Society of Anaesthesiology. Eur J Anaesthesiol.

[3] Dobson G., Chow L., Filteau L. (2021). Guidelines to the practice of anesthesia — revised edition 2021. Can J Anaesth.

[4] Marcucci C., Seagull F.J., Loreck D., Bourke D.L., Sandson N.B. (2010). Capacity to give surgical consent does not imply capacity to give anesthesia consent: implications for anesthesiologists. Anesth Analg.

[5] Yentis S.M., Hartle A.J., Barker I.R. (2017). AAGBI: consent for anaesthesia 2017: association of anaesthetists of great Britain and Ireland. Anaesthesia.

[6] Fung D., Cohen M. (2001). What do outpatients value most in their anesthesia care?. Can J Anaesth.

[7] Stubenrouch F.E., Mus E.M.K., Lut J.W., Hesselink E.M., Ubbink D.T. (2017). The current level of shared decision-making in anesthesiology: an exploratory study. BMC Anesthesiol.

[8] Sturgess J., Clapp J.T., Fleisher L.A. (2019). Shared decision-making in peri-operative medicine: a narrative review. Anaesthesia.

[9] Tait A.R., Teig M.K., Voepel-Lewis T. (2014). Informed consent for anesthesia: a review of practice and strategies for optimizing the consent process. Can J Anaesth.

[10] Chrimes N., Marshall S.D. (2018). The illusion of informed consent. Anaesthesia.

[11] Kain Z.N., Vakharia S., Garson L. (2014). The perioperative surgical home as a future perioperative practice model. Anesth Analg.

[12] van den Berg A.M.A., Stalmeier P.F.M., Scheffer G.J., Hermens R.P., Bucx M.J.L. (2019). Shared decision-making for postoperative analgesia: a semistructured qualitative study. Eur J Anaesthesiol.

[13] Waisel D.B. (2011). Let the patient drive the informed consent process: ignore legal requirements. Anesth Analg.

[14] Asehnoune K., Albaladejo P., Smail N. (2000). [Information and anesthesia: what does the patient desire?]. Ann Fr Anesth Reanim.

[15] Bethune A., Davila-Foyo M., Valli M., e-Consent da Costa L. (2018). Approaching surgical consent with mobile technology. Can J Surg.

[16] Southerland W.A., Beight L.J., Shapiro F.E., Urman R.D. (2020). Decision aids in anesthesia: do they help?. Curr Opin Anaesthesiol.

[17] Urman R.D., Southerland W.A., Shapiro F.E., Joshi G.P. (2019). Concepts for the development of anesthesia-related patient decision aids. Anesth Analg.

[18] Webster F., Bremner S., McCartney C.J. (2011). Patient experiences as knowledge for the evidence base: a qualitative approach to understanding patient experiences regarding the use of regional anesthesia for hip and knee arthroplasty. Reg Anesth Pain Med.

[19] Wilbanks J. (2018). Design issues in e-consent. J Law Med Ethics.

[20] Bollschweiler E., Apitzsch J., Obliers R. (2008). Improving informed consent of surgical patients using a multimedia-based program? Results of a prospective randomized multicenter study of patients before cholecystectomy. Ann Surg.

[21] Mawhinney G., Thakar C., Williamson V., Rothenfluh D.A., Reynolds J. (2019). Oxford Video Informed Consent Tool (OxVIC): a pilot study of informed video consent in spinal surgery and preoperative patient satisfaction. BMJ Open.

[22] Cyna A.M., Simmons S.W. (2017). Guidelines on informed consent in anaesthesia: unrealistic, unethical, untenable. Br J Anaesth.

[23] Joseph-Williams N., Elwyn G., Edwards A. (2014). Knowledge is not power for patients: a systematic review and thematic synthesis of patient-reported barriers and facilitators to shared decision making. Patient Educ Couns.

[24] Klafta J.M., Roizen M.F. (1996). Current understanding of patients’ attitudes toward and preparation for anesthesia: a review. Anesth Analg.

