# Supplementary material for: Autonomous patient consent for anaesthesia without preoperative consultation: a qualitative feasibility study including low-risk procedures
Source: BJA Open. 2022 Jul 13;3:100022. doi: 10.1016/j.bjao.2022.100022 (PMC10430827; doi:10.1016/j.bjao.2022.100022)
Supplement: Multimedia component 1 [file mmc1.docx]

**Appendix A. Topics adressed in the web-based digital application, choices for patients and matching questions from the interview guide.**

| **Web-**  **page** | **Title**  **(with basic information on every page)** | **Subtitles (after clicking additional information appears)** | **Choices for patients** | **Interview guide - main questions** |
| --- | --- | --- | --- | --- |
| 1 | Introduction | None | Start of e-consult or stop program and make an appointment | Do you know why you have to visit the anaesthesiologist?  Wat will be discussed?  You have consented for surgery, does it include anaesthesia consent?  What do you want to discuss with the anaesthesiologist? |
| 2 | Login by secured access |  | Secured login | Do you use secured access  Is it safe?  Can the anaesthesiologist trust the safety of your e-consult? |
| 3 | Conduct of anaesthesia (general anaesthesia/ sedation) | What is general anaesthesia/sedation?  What happens to me during anaesthesia/sedation?  What happens to me between arrival at the OR and the end of anaesthesia? | Choice for additional information in subtitles  Choice for film about anaesthesia | What do you think of the information provided?  Why do you (not) click for more information?  If a patient had anaesthesia before: How does this information relate to your previous experiences?  Do you have enough information at this moment to consent? |
| 4 | Recovery ward | Am I awake at the recovery ward?  Can I have visitors at the recovery ward?  When can I go to the general ward or home? | Choice for additional information in subtitles  Choice for film about recovery ward | What do you think of the information provided?  Why do you (not) click for more information?  If a patient had anaesthesia before: How does this information relate to your previous experiences?  Do you have enough information at this moment consent? |
| 5 | Minor side effects | Nausea  Pain  Sore Throat | Choice for additional information in subtitles | What do you expect with regard to side effects?  How much do you want to know about side effects?  How does this information makes you feel?  Why do you (not) click for more information?  If a patient had anaesthesia before: How does this information relate to your previous experiences?  How does this information relates to your current planned surgery?  Do you have enough information at this moment consent? |
| 6 | Severe but rare complications | More about rare complications  Death  Dementia  Awareness  Aspiration | Choice for additional information in subtitles | About title page: What do you think after reading the title?  How much do you want to know about complications?  Why do you (not) click for more information?  How does this information makes you feel?  If a patient had anaesthesia before: how does this information relate to your previous experiences?  How does this information relates to your current planned surgery?  Does this information influence your consent?  Do you have enough information at this moment consent?  Do we have to show this information to patients?  Currently the anaesthesiologist initiates the complications to be discussed. How do you think about this? |
| 7 | Preparing for anaesthesia |  |  |  |
| 8 | Fasting policy |  | Choice for film about fasting  Choice for signing if preparation is understood (yes, no, doubt) | What do you think about this topic?  If you click yes, can we hold you responsible, if you do not follow the fasting preparations? |
| 9 | Continuation of medication |  | Choice for film about medication continuation/cessation  Choice for signing if preparation is understood (yes, no, doubt) | What do you think about this topic? |
| 10 | Getting help during the first night at home |  | Choice for film about help  Choice for signing if preparation is understood (yes, no, doubt) | What do you think about this topic? |
| 11 | What to do if you get sick the week before your anaesthesia |  | Choice for film about getting sick  Choice for signing if preparation is understood (yes, no, doubt) | What do you think about this topic? |
| 12 | Blood transfusion | More about transfusion  Advantages Disadvantages  Additional information | Choice for additional information in subtitles  Choice for consent for transfusion (yes, no, doubt) | What do you think about this topic?  Did you know you had to consent for transfusion?  Why do you (not) click for more information? |
| 13 | Informed consent | I understand what will happen to me  I have gotten enough information about side effects and risks of anaesthesia  I consent for anaesthesia  I understand what preparations I have to do  I have a question | Choice for consent (yes, no, doubt)  Choice for consent (yes, no, doubt)  Choice for consent (yes, no, doubt)  Choice for consent (yes, no, doubt)  Free text field and option to choose between: I will ask it at the day of surgery, I will send an e-consult, I want a consultation by telephone, I want a consultation in the hospital | Do you have enough information to give consent?  If yes, why? If no/doubt, why not?  Do you feel barriers to ask questions?  Currently a consultation with an anaesthesiologist is needed for official consent. This e-consent can be an alternative. How do you think about this? For who or under which circumstances would e-consent be suitable?  When do you need an consultation with an anaesthesiologist? |
| 14 | What happens after this e-consent | Patient will receive a letter within 3 days with confirmation and approval of anaesthesiologist or appointment if necessary |  | What do you think of this information? |
| 15 | End of program |  |  | Additional questions:  What do you think of e-consenting?  Would you recommend this to others?  What are advantages and disadvantages of e-consenting at home?  When do you want an consultation with an anaesthesiologist?  Does the COVID-19 pandemic influence your choice of e-consulting versus consultation in the hospital?  During consultation with an anaesthesiologist communication and consent can be at a high pace. How is this for you?  Some people are afraid of anaesthesia. How is this for you?  How much do you want to know about your planned anaesthesia |

**Appendix B. Short questionnaire.**

| Wat is your age? |
| --- |
| What is your sex? |
| What is your highest level of education? |
| Digital skills: Do you use a computer/tablet/smartphone for*   - Buying goods - Banking - Taxes - Insurance - Access to personal health record - Email - Work - Social media - Leisure - To get information about any topic of interest - To get information about my health |
| Do you search for information about your own health/sickness/surgery? |
| How do you get this information?   - Internet - Personal health record - Friends/family - Other |
| How much do you want to know about upcoming anaesthesia?   - Nothing - As less as possible - Enough to know what will happen to me and to make a decision - As much as possible - Other |
| Wat (surgical) procedure will you get? |
| Do you have previous experience with anaesthesia? |
| If yes, which one(s)   - General anaesthesia - Procedural sedation - Neuraxial anaesthesia (spinal or epidural) - Peripheral nerve block |
| Have you looked for information about anaesthesia before you came to the clinic or did you already have information |
| Where did you get this information from?   - Internet (google, wikipedia) - Official website from the hospital - Paper leaflets - From referring physician - My own previous experiences - Other |

**Appendix C. Code book.**

| **Codes and subcode** | Description |
| --- | --- |
| First choice | What chooses the interviewee intuitively: application or consultation? |
| consultation |  |
| application |  |
| Recommend application to others | Would the interviewee recommend the application to others? |
| Yes |  |
| No |  |
| Doubt |  |
| Consultation versus application | Advantages and disadvantages of application and consultation. |
| Consultation | Advantages and disadvantages of consultation. |
| Application | Advantages and disadvantages of application. |
| Importance of anaesthesia versus surgery |  |
| Bloodtransfusion | Questions and answers related to the topic blood transfusion. |
| Consent bloodtransfusion | Code for all reports about consent for blood transfusion. |
| Information bloodtransfusion | Code for all reports about information about transfusion: what did interviewees already knew about transfusion, why do they read information, previous experiences. |
| COVID-19 | All COVID related reports. |
| Worries about Covid | Afraid of COVID. |
| No worries Covid | No fears for CoOVID. |
| Personal secured access system | All reports about secured access system to personal health record. |
| Trust in secured access | Can the anesthesiologist entrust a patient that he does not fraud with the secured access? |
| Secured access safety | Is secured access safe, for example can it be hacked? |
| Uses secured access | Does the interviewee use secured access. |
| Barriers | Are there barriers that prevent interviewees from contacting an anaesthesiologist or hospital. For example: I don’t want to call the hospital, because they are busy. |
| Emotions | All reports and observations of emotions. |
| Fear |  |
| Anxiety |  |
| Experience | Previous experiences of interviewees with hospitals, surgery, other experiences that influence them in need for information and consenting. |
| Own experiences | Own experiences with anaesthesia. |
| Experiences from others | Experiences from others with anaesthesia. |
| Negative experiences |  |
| Neutral experiences |  |
| Positive experiences |  |
| Comforting | Code for all (implicit) reports about comforting. |
| Informed consent | Reports from interviewees about information provisioning and consent. |
| Consent | Every report about consenting. |
| Choice | Do interviewees have the feeling that they have a choice in the decision for anaesthesia? |
| Timing of consent | After which webpage does the interviewee report that he has enough information to consent? |
| Severe complications | Reports about the webpage severe complications. |
| Reports about information provisioning | All reports about information provisioning. |
| Learns from new information |  |
| Not reading additional information |  |
| Enough information | Did interviewee have enough information? |
| Reading additional information |  |
| Application | Report about how to improve the application. |
| Unclear | Reports about topics that an interviewee doesn’t understand. |
| Does researcher think patient can use the application | Does the research think that the patient knows enough for a valid consent? |
| Personality | Reports about personality of interviewee. |
| Tasks of anaesthesiologist | What should an anaesthesiologist do during consultation? |
| Discuss the conduct of anaesthesia and discussion about risks |  |
| Physical examination |  |
| Being a ‘figurehead of anaesthesia’ |  |
| Safety | Examine if a patient is healthy enough to survive anaesthesia. |
| Responsibilities patient | Every report about responsibilities of patients. Can anesthesiologist entrust the patient to take his responsibilities? |
| Using secured access |  |
| Reading the offered information |  |
| Responsible for correct preparations for anaesthesia |  |
| Wrongly understood by patient | Information that is misinterpreted by interviewees. |
| Faith and trust | Where interviewees report about faith (in general, god, healthcare, etc.) |
| Wanting to see a familiar face | Reports about wanting to see an anaesthesiologist before the surgery, because knowing some-one gives hope and comforting |
| Faith that everything will be fine | ‘blind faith’ |
| Faith in physicians |  |
| Referring to other people | Reports from interviewees about other patients. For example: I could use the application, but by aged neighbor could not. |
| Those who consider secured access safe |  |
| Anxious patients |  |
| Major surgery or comorbidity |  |
| Low digital skills |  |
| Age |  |
| Those who fraud with secured access |  |
| Patients with previous experience |  |
| Patients who want to know much |  |
| Patients who do not speak up for themselves |  |
| Patients who do not want to know anything |  |
| Unexperienced patients |  |
| Patients who do not read of speak Dutch |  |
| Other |  |
| Questions & answers | Does interviewees have questions after following the application? How do they want to receive their answer? How soon do they want their answer? |
| Answer on day of surgery, earlier not necessary |  |
| By phone |  |
| By email |  |
| By consultation |  |
| No clear answer |  |
| As soon as possible |  |
